# Individual and Combined Effect of Zinc-L-Selenomethionine Complex with Mannan-Oligosaccharide on Growth Performance, Carcass Characteristics, Gut Development and Immune Response in Broilers

**DOI:** 10.3390/vetsci12080768

**Published:** 2025-08-17

**Authors:** Hammad Talib, Ehsaan Ullah Khan, Muhammad Muneeb, Abdul Mateen, Saima Naveed, Jibran Hussain, Sohail Ahmad, Elham Assadi Soumeh, Abdulkareem M. Matar

**Affiliations:** 1 Department of Animal Nutrition, Faculty of Animal Production and Technology, University of Veterinary and Animal Sciences, Lahore 54000, Pakistan; hammadtalib15@gmail.com (H.T.); ehsaan@uvas.edu.pk (E.U.K.); 2022-mphil-1331@uvas.edu.pk (M.M.); saimamahad@uvas.edu.pk (S.N.); 2 School of Agricultural Technology and Food Industry, Walailak University, Nakhon Si Thammarat 80160, Thailand; abdulmateen2016786@gmail.com; 3 Department of Poultry Production, Faculty of Animal Production and Technology, University of Veterinary and Animal Sciences, Lahore 54000, Pakistan; jibran.hussain@uvas.edu.pk; 4 School of Agriculture and Food Sustainability, The University of Queensland, Gatton, QLD 4343, Australia; e.assadisoumeh@uq.edu.au; 5 Department of Animal Production, College of Food and Agriculture Sciences, King Saud University, P.O. Box 2460, Riyadh 11451, Saudi Arabia

**Keywords:** antibiotic alternative, broiler, gut health, mannan-oligosaccharide, selenium

## Abstract

Due to the increased restrictions on antibiotic growth promoters (AGPs), natural substitutes such as organic selenium and prebiotics are gaining increased attention to enhance the gut health and performance of broilers. This study aimed at assessing the effects of organic selenium separately and in combination with mannan-oligosaccharides (MOSs) on gut development, immunity, carcass traits and overall growth performance in broiler chickens. For this study, 528-day-old straight-run broiler chicks (Ross-308) were assigned to four dietary treatments (six replicates of 22 birds each), in a completely randomized design under a 2 × 2 factorial layout, including: (1) Se0.2 (0.20 mg/kg organic selenium), (2) Se0.2 + MOS (0.20 mg/kg selenium + 1 g/kg MOS), (3) Se0.4 (0.40 mg/kg selenium) and (4) Se0.4 + MOS (0.40 mg/kg selenium + 1 g/kg MOS). The results showed that the lower level of organic selenium, along with MOS (Se0.2 + MOS), resulted in significantly enhanced (*p* ≤ 0.05) feed conversion ratio, body weight gain and livability. Whereas the higher level of organic selenium, along with prebiotic (Se0.4 + MOS), remarkably (*p* ≤ 0.05) improved carcass traits, immunity and villus height in broilers. In conclusion, the combined provision of 0.4 mg/kg organic Se and 1 g/kg MOS optimally enhances broiler performance, immunity and gut health, offering a promising substitute to AGPs.

## Simple Summary

Due to ongoing restrictions on the use of antibiotic growth promoters (AGPs) in the poultry industry, natural substitutes like organic selenium and prebiotics are becoming more popular. The present study examined the effects of organic selenium separately and in combination with mannan-oligosaccharides (MOSs) on broiler growth performance, gut development, carcass characteristics and immunity. In this study, 528 broiler chickens were studied under four dietary treatments, including two organic selenium inclusion levels (0.2 or 0.4 mg/kg) with or without MOSs (1 g/kg). The results indicated that the combination of lower selenium levels with MOSs resulted in enhanced body weight gain (BWG) and feed efficiency. In contrast to that, higher selenium levels with MOS resulted in significantly enhanced carcass quality, gut morphology and immune response. Overall, dietary treatment containing 0.4 mg/kg organic selenium and 1 g/kg MOS was found to be most effective and beneficial for enhancing broiler growth and productivity. The obtained results demonstrate the potential of the simultaneous use of organic selenium and MOS as an effective natural alternative to antibiotics in poultry nutrition.

## 1. Introduction

The global rise in population has necessitated rapid and compelled advancements in the food sector worldwide, leading to significant challenges in ensuring adequate food supply for all individuals on the planet. All over the world, the poultry industry is playing an imperative role in the national income of many countries. Poultry meat also provides an economic and affordable source of animal protein to many low-income families to overcome their protein deficiency. The primary objectives of controlled systems for broiler production are to cultivate healthy broilers that reach target body weight rapidly. There has been increasing research into alternative methods to improve development, boost immunity and promote overall health for more efficient bird growth [1,2].

Antibiotics have been used widely in poultry production due to their ability to increase weight gain, decrease colonization of pathogens in the gut and improve feed efficiency [3]. Increasing concerns about antibiotic resistance in bacteria with implications for human and animal health have resulted in reduced usage of antibiotic growth promoters (AGPs) [4]. Moreover, the demand for antibiotic-free (ABF) food has considerably increased in this era [5]. Due to these concerns, the European Union has prohibited the use of AGPs [6]. In the United States, AGPs use is now restricted under the Veterinary Feed Directive [7]. Supplementation strategies may contribute to reducing reliance on AGPs. Hence, there is an increasing interest in searching for alternatives to AGPs in the poultry industry [8].

Minerals are considered to have an outstanding role in the growth, immunity and health of broiler chickens. Selenium (Se) is a vital and essential micro-mineral that is required in the diet of broilers. Its supplementation can be made possible either by inorganic sources, like sodium selenite, or with organic molecular complexes like selenomethionine; however, the two selenium sources have different utilization efficiencies. Selenium from organic sources shows much higher retention in muscle tissue along with higher antioxidant properties and bioavailability as compared to the inorganic sources [9]. Additionally, Se in its organic form is relatively less toxic in nature and more environmentally friendly. Performance traits of broilers like feed intake, body weight gain (BWG) and feed conversion ratio (FCR) have been reported to be higher when birds were fed with organic Se in comparison to its inorganic forms [10]. Selenium is an influential antioxidant with a remarkable role in many vital metabolic pathways, including immunity [11], antioxidant defense systems [12] and metabolism of thyroid hormone [13]. Selenium deficiency in broilers’ diet is quite hazardous, and many studies signify its appropriate inclusion. Depressed BWG, immunity and thyroxin, as well as increased oxidative diathesis and pancreatic fibrosis, are among the common manifestations of Se deficiency in broilers [11,14]. Moreover, deficiency of Se can harm the cellular and humoral immunity by deactivation of B-cells, which leads to a reduction in immunoglobulin levels. Selenium and vitamin E in combination may also influence the propagation of lymphoid cells, immune complexes and levels of circulatory immunoglobulins, which would improve antibody performance [15]. On the contrary, overdosing of Se is also a critical issue in broilers, resulting in the inhibition of the activity of several enzymes like choline oxidase, tyramine and d-proline oxidase [16]. Both of the symptoms of shortage and overdose can be reverted by the use of the recommended provision of Se in the diet of broilers from an appropriate source.

Prebiotics are mainly oligosaccharides that are mannan-oligosaccharides (MOSs), galacto-oligosaccharides, isomalto-oligosaccharides, fructo-oligosaccharides (FOSs), chit oligosaccharides and xylo-oligosaccharides [17]. Mannan-oligosaccharides are obtained from yeast (*Saccharomyces cerevisiae*) and contain glucans, phosphate radicals and mannose [18]. Prebiotics hold a significant reputation due to boosting the development of intestinal tissue, microflora establishment, humoral immunity buildup, and enhancing growth performance [19]. Prebiotics considerably reduce the pathogenic microbiota in the gut and help in the enhancement of broilers’ immunity [20]. Prebiotics stimulate the colonization and proliferation of beneficial gut microflora in broilers, supporting intestinal health and function [1]. According to Hooge [21], the supplementation of prebiotics in the broiler diet may improve the growth performance. Numerous studies also demonstrate that prebiotics significantly affect mineral absorption by decreasing the intestinal pH [22,23,24].

According to recent research, precise supplementation of prebiotics and important trace minerals may serve as a potential replacement for AGPs while maintaining the production performance and immunological competence in broilers [25]. The concurrent use of functional feed additives like prebiotics and supplements with higher bioavailability (i.e., organic selenium) is gaining popularity nowadays as a strategy to boost broiler health and productivity. Selenium is widely recognized for its involvement in immunological function, antioxidant defense and general performance, especially in its organic form, such as zinc-L-selenomethionine [26,27,28]. Similarly, MOSs are popular prebiotics, well known to enhance intestinal structure and beneficial bacteria, thus improving gut health [29,30,31,32]. In poultry production, both additives have shown individual efficacy; however, the studies assessing their combined effects remain limited. Because of their distinct but possibly complementary modes of action, a synergistic effect higher than the individual effect of either of them is possible when used together. Thus, the purpose of this study was to explore the impact of dietary zinc-L-selenomethionine individually or in combination with MOS on growth, carcass traits, gut development and immune response in broilers. It was hypothesized that the combined supplementation would result in greater improvements in these parameters than organic selenium alone. Addressing this knowledge gap is important for optimizing nutritional strategies that can not only help support broiler performance and health but also reduce reliance on AGPs, thereby contributing to safer and more sustainable poultry production.

## 2. Materials and Methods

### 2.1. Ethical Approval

The study was organized in an environment controlled, floor-rearing “Broiler Experimental House”, University of Veterinary and Animal Sciences (UVAS), Ravi Campus, C block, Pattoki, Pakistan, for 35 days. A prior consent was acquired from the “Ethical Review Committee” of UVAS, Lahore (Approval Number: 214; Date: 5 April 2021).

### 2.2. Experimental Design

In total, 528-day-old straight-run broiler chicks (Ross-308) were placed in a completely randomized design (CRD) with a 2 × 2 factorial layout comprising four dietary treatments (six replications of 22 birds each): (1) Se 0.2 (0.20 mg/kg organic Se), (2) Se 0.2 + MOS (0.20 mg/kg selenium + 1 g/kg MOS), (3) Se 0.4 (0.40 mg/kg selenium) and (4) Se 0.4 + MOS (0.40 mg/kg selenium + 1 g/kg MOS) (Table 1). The organic Se supplement (Availa^®^ Se 1000, Zinpro^®^ Corporation, Eden Prairie, MN 55344, USA) contains 0.1% zinc-L-selenomethionine with calcium carbonate, silicon dioxide, and mineral oil as carriers, while the MOS (Bio-Mos^®^, Alltech Inc.; Islamabad 44000, Pakistan) was derived from a selected *Saccharomyces cerevisiae* strain.

### 2.3. Bird Husbandry

During this study, all birds were managed following the management guidelines of Ross-308 [33]. Briefly, the broiler house was prepared by washing with tap water, disinfecting with bleaching powder, and fumigating with a formalin and KMnO_4_ solution prior to chick placement. Initially, the house temperature was 34 °C and later on reduced by 2.8 °C weekly until day 21. The lighting period was 23 h throughout the trial. Rice husk was used as a litter material 3–4 inches depth. Water lines were sanitized and adjusted to bird eye level, with two manual feeders provided per replicate. Standard vaccination protocols were followed against coccidia and common viral diseases.

Feed and fresh water were offered *ad libitum* for the whole experiment. For the first seven days, the feed was offered in specially designed trays, and then onwards in round feeding bins. Broiler diets, formulated as per Ross-308 standards [34], were offered for the starter phase (1–21 d) and finisher phase (22–35 d) (Table 2). The starter diet was prepared as 2 mm crumbles, and the finisher diets as 4 mm pellets. All basal diets were isoproteic and isoenergetic, without the addition of AGPs and coccidiostats. Fresh experimental diets were produced for each phase. Chemical composition of feed ingredients and mixed feed was determined using proximate analysis by following the standard methods of AOAC [35].

### 2.4. Parameters Studied

#### 2.4.1. Growth Performance

The growth performance was assessed as previously explained by Yameen et al. [37]. Weekly feed intake was determined by deducting the remaining feed from the total amount of feed provided to each replicate.

Feed intake (g) = Feed offered to birds (g) − Feed refused by the birds (g)

At the beginning of the experiment, the initial weight of chickens was noted individually. The BWG was calculated weekly by deducting the initial weight from the final weight.

BWG (g) = Final weight (g) − Initial weight (g)

Feed conversion ratio (FCR) was calculated weekly by applying the given formula:

FCR = Feed consumed (g)/Body weight gain (g)

Mortality was noted every day (if any) for all treatments, and livability was computed by using the given formula.

$$Livability\;\left(\%\right)=\frac{Number\;of\;live\;birds}{Total\;birds\;placed}\times 100$$

#### 2.4.2. Carcass Traits

When the experiment was finished, carcass evaluation was done by randomly selecting four birds from each replicate for slaughtering. Birds were slaughtered humanely cutting the jugular vein and carotid artery by following the Islamic/Halal method. Each bird was separated into parts for carcass evaluation as previously described by Ojewola et al. [38].

Head, shanks and visceral organs were taken out, followed by the weight of the carcass that remained, and then the dressing percentage was computed through the following formula:

$$Carcass\;yield\;(\%)=\frac{Carcass\;weight\;(g)}{Live\;body\;Weight\;(g)}\times 100$$

Breast yield percentage was computed using the following formula:

$$Breast\;yield\;(\%)=\frac{Whole\;breast\;weight\;(g)}{Carcass\;weight\;(g)}\times 100$$

Leg percentage was recorded by using the formula given below:

$$Leg\;quarter\;yield\;(\%)=\frac{Leg\;weight\;(g)}{Carcass\;Weight\;(g)}\times 100$$

Giblets (heart, liver and gizzard) were weighed and expressed on a percent basis.

$$Giblets\;(\%)=\frac{Giblets\;weight\;(g)}{Live\;body\;weight\;(g)}\times 100$$

#### 2.4.3. Immune Status

At the completion of the trial, blood samples from 3 birds/replicate were collected from the wing veins in evacuated tubes and serum was harvested from the collected samples. To obtain serum, blood samples were left at room temperature for 1 h, and then centrifugation (Beckman J25I; Beckman Instruments, Inc., Diagnostics Division Headquarters 250 South Kraemer Boulevard, Brea, California, USA) was performed at 1500× *g* at 4 °C for 20 min. The obtained serum was separated into aliquots and kept at −20 °C for antibody titer analysis against Newcastle disease virus (ND). Antibody titer against NDV was evaluated on the serum samples through hemagglutination and hemagglutination–inhibition (HI) tests following Muneeb et al. [8]. During slaughtering, the bursa and spleen were also collected and weighed.

#### 2.4.4. Intestinal Morphology

Intestinal tissues were also collected from the same birds sampled for blood and carcass. Briefly, a 2 cm part of the small intestine from the duodenum (distal to the duodenal loop) was taken. The luminal segment of intestinal samples was flushed with normal saline and fixed in formalin (10%) for 48 h. The preserved samples were then washed and dehydrated in various concentrations of alcohol and embedded in paraffin wax. From each sample, 5 μm thick tissue sections were cut by a microtome and mounted on a glass slide. The standard staining procedure was performed using hematoxylin and eosin. A light microscope was utilized to observe the villus height and crypt depth, while software (Pixel Pro^®^ 3.2™) was used to record the histomorphological measurements, as previously explained [36].

#### 2.4.5. Statistical Analysis

Two-way ANOVA for factorial design was carried out to analyze the data statistically using PROC GLM in SAS software (version 9.1). Least square means were compared using, DMRT (Duncan’s Multiple Range Test), considering a significance level at *p* ≤ 0.05.

## 3. Results

### 3.1. Growth Performance

The effects of selenomethionine with or without MOS on the growth performance of broilers are shown in Table 3. Feed intake during starter and finisher phases, and in the overall period, was notably (*p* ≤ 0.05) affected by selenomethionine and MOS. The higher (*p* ≤ 0.05) FI was recorded in the birds offered with only Se (Se0.2 and Se0.4), while the lowest (*p* ≤ 0.05) FI was noted in the Se0.4 + MOS during the starter phase. The same pattern was observed during the finisher phase and in the overall (1–35 d) duration.

These findings revealed a significant (*p* ≤ 0.05) synergy between the selenomethionine and MOS addition in the diet on BWG of broiler chicken in the starter phase, finisher phase and overall period. Treatments supplemented with MOS during the starter phase showed the highest BWG. However, during the finisher phase (22–35 d) and overall period (1–35 d), the highest BWG was observed in Se0.2 + MOS. The effect of selenomethionine and MOS and their interaction (Se × MOS) remained significant (*p* ≤ 0.05) for the FCR during the starter (1–21 d), finisher (22–35 d) and overall period (1–35 d). The Se0.2 + MOS depicted the best FCR as compared to other treatments during the finisher and overall duration. Similarly, the effect of Se alone and its combination with MOS was found significant (*p* ≤ 0.05) for the livability of broilers during the whole trial (1–35 d), and the highest value was found in Se0.2 + MOS among all the treatments.

### 3.2. Carcass Characteristics

The effects of selenomethionine at 0.2 mg/kg and 0.4 mg/kg with or without MOS on carcass characteristics of broiler are shown in Table 4. Selenomethionine at a greater level with MOS (Se0.4 + MOS) resulted in significantly higher carcass weight and carcass yield (*p* ≤ 0.05) as compared to other treatments. A non-significant (*p* > 0.05) impact of either Se or MOS, as well as their interaction (Se × MOS), was observed for the heart weight. The liver and gizzard weights were found to be higher (*p* ≤ 0.05) in Se 0.4 and Se0.4 + MOS as compared to other treatments. Treatment with Se0.4 + MOS supplementation showed the highest leg quarter weight and breast weight (*p* ≤ 0.05).

### 3.3. Immunity

**Figure 1:**
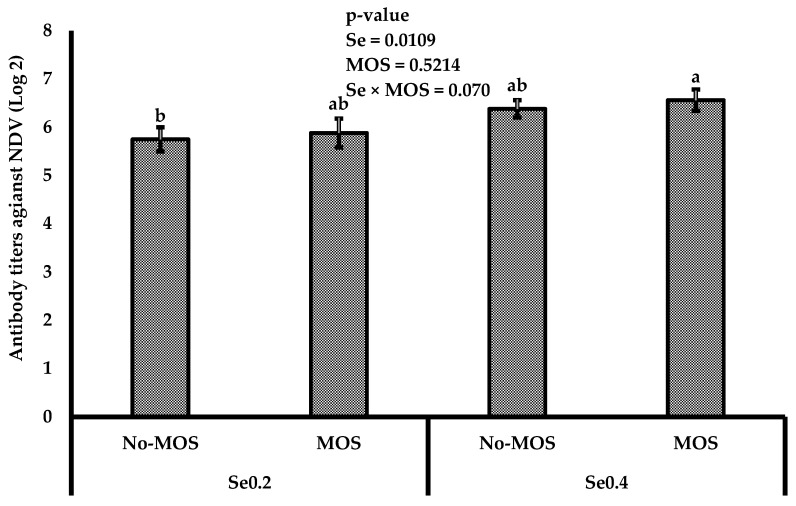
Impact of dietary treatments on HAHI antibody titers against NDV on day 35 in broilers. The lowercase letters represent the significant differences acorss treatments.

The effect of organic Se, along with MOS, on the development of immune organs is expressed in Table 5. A significant (*p* ≤ 0.05) interaction between selenomethionine levels with or without MOS on the development of immune organs on day 35 was observed. The broilers fed with Se0.4 + MOS have the highest (*p* ≤ 0.05) weight of bursa and spleen in comparison to other treatments. Moreover, a significant (*p* ≤ 0.05) interaction among selenium levels and the presence of MOS for NDV titer was observed at day 35, where Se0.4 + MOS showed the highest antibody titers against NDV (Figure 1).

### 3.4. Gut Morphology

**Figure 2:**
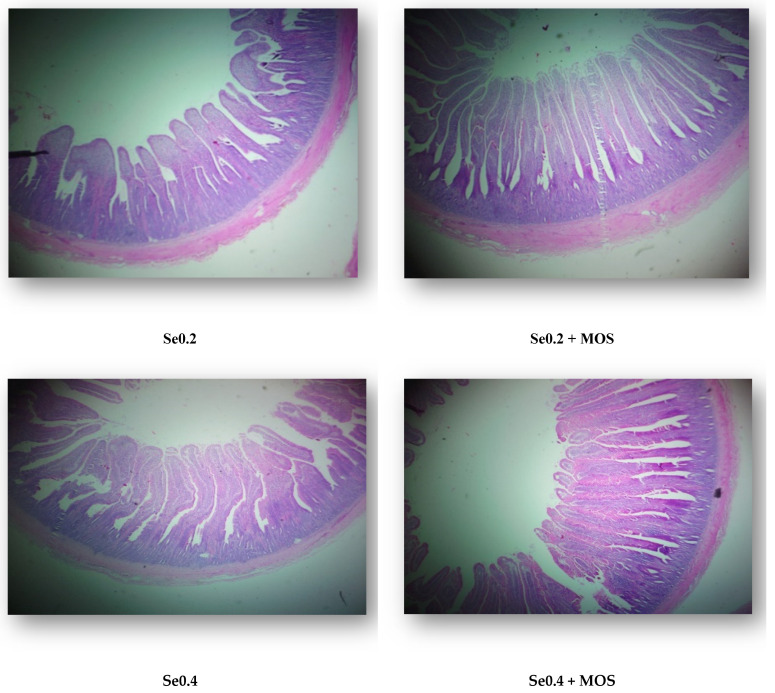
Infographics showing the duodenal morphology of broiler chickens in various treatments (day 35).

The effect of selenomethionine with or without MOS supplementation on the histomorphological traits of the duodenum is depicted in Table 6. There was no significant effect (*p* > 0.05) of Se levels, and its combination with MOS on all the histomorphological traits: villus height, crypt depth and the ratio of VH to CD (Figure 2).

## 4. Discussion

### 4.1. Growth Performance

Selenium has a complex metabolism, and it varies depending on the basis of its sources. The form of Se influences its absorption, retention and subsequent utilization. In this experiment, the interaction of selenomethionine and MOS was significant for FI, BW and FCR in all the inclusion levels during the period of the experiment (1–35 d). Our results are in accordance with the findings of Wang et al. [39], who mentioned that the chicks, when fed with Se 0.2 and 0.4 mg/kg Se-supplemented diet, had higher FI. The reason may be that Se is among the essential trace elements carrying out important functions in animals, and it also serves as a crucial part of selenoproteins. Selenium is also a cofactor and activator of 5’ deiodinase, a crucial enzyme of tri-iodothyronine (T3) synthesis, and T3 regulates protein assimilation and energy in the body, which is how it controls animal growth, especially in birds. Consequently, Se supplementation in the chicken diet could improve their growth performance, perhaps as a result of improved energy utilization and protein digestibility [40,41]. The outcomes of our study are consistent with those of Bakhshalinejad et al. [42], who reported that chicks fed diets with organic Se at a level of 0.4 mg/kg significantly improved the daily FI of broilers. Similar outcomes were observed by Yang et al. [10] and explained that fortification of organic selenium at 0.3 mg/kg in the broiler diet had significantly increased the FI compared with the same level of supplementation of inorganic selenium. Abdel Hafeez et al. [31] reported that MOS treatment resulted in increased feed intake than the control group (which did not contain prebiotics) in broilers. Probiotics act by managing the dynamic equilibrium of microbiota in the gut [43] and function by specifically boosting the proliferation and activity of useful bacteria [44,45]. These advantageous effects may decrease digestive issues and strengthen the overall health and well-being of the host animal. Since healthy animals effectively use and transform nutrients of feed into constant growth, therefore, the positive impacts of prebiotics on intestinal microbiota may result in improved BWG and feed conversion. Numerous studies have demonstrated that nutrient digestion and absorption are among the main underlying factors for improved growth of birds with prebiotics [46,47]. Additionally, the useful bacteria such as *Lactobacillus* spp. synthesize digestive enzymes that may aid in improving the digestive and feed conversion processes in the host animal. In fact, when poultry birds were given prebiotics, there were seen improvements in the digestion of ileal nutrients, retention of nitrogen, villus height [48], colonization of *Bifidobacterium* spp., *Lactobacillus* spp. [49] and a decrease in *Salmonella* prevalence [50,51]. Although it has been widely documented that prebiotic usage alters GI health and decreases the pathogen load, the process underlying this effect is unclear. The increased FI might be the result of biological feed additives that are changing the pH of the intestine, which alters the microbial population and the absorption of nutrients, hence boosting feed utilization efficiency [43]. The decrease in mortality was ascribed to the ability of these additives to suppress the microorganisms in the gut by altering the pH. Furthermore, feed additives have the ability to enhance intestinal length and villus height, providing a larger surface area for better nutrient absorption [52]. Our outcomes also aligned with the findings of Abudabos et al. [53], who determined a significantly increased BWG when broilers fed on prebiotics (0.75 g/kg) than the control diet (no supplementation) (1922 g vs. 1660 g from 15 to 42 days). The outcomes of the present results regarding BWG and FCR also corroborate with the outcomes of Shahir et al. [54], who mentioned better FCR in broiler birds fed a diet containing MOS. Soumeh et al. [29] shared that a significantly improved FCR (1.70) was seen in broilers supplemented with prebiotics (2 g/kg) as compared to the control diet (no supplementation) (1.72), as observed in our findings. According to the literature, the impact of prebiotics on growth performance may be related to metabolic alterations that increase the activity of digestive enzymes [55], enhanced FI and digestion, reduced bacterial enzyme activity, and reduced ammonia generation [56]. However, variables such as bird age, sex, breed, general farm hygiene, environmental stress, type, concentration and dosage, administration methods and frequency may be the cause of discrepancies in research reports [57].

### 4.2. Carcass Traits

The poultry industry aims to produce a higher yield or dressing percentage, saleable products and, as a result, a higher edible portion. Inclusion of feed additives generally has the potential to speed up the metabolism rate and ultimately enhance the size of internal organs, as also observed here in our study. In our study, the interaction of selenomethionine and MOS was significant for the carcass traits (except heart weight) among all the treatments. These outcomes agree with the results of Choct et al. [9], who concluded that the supplementation of organic selenium significantly increased eviscerated weight. Notably, organic Se improved eviscerated weight, and it may be due to an enhancement of water retention and protein deposition in tissues. A significant interaction existed among the breast fillet weight, source and the concentration of Se. Current findings support the outcomes of Whanger and Butler [58] and Mahan and Kim [59], who reported a better retention of organic Se in animal muscle. Selenized yeast contains selenomethionine that shows instant absorption by proteinaceous tissue in place of its sulfur counterpart methionine. The findings of our study on the heart weight are in line with the outcomes of Rao et al. [60], who reported a non-significant effect on the broilers’ giblet weight that may be due to feeding a precise diet with adequate nutrient levels. We noted a substantial elevation in liver weight in birds administered 0.4 mg/kg Se (with and without MOS) relative to those receiving 0.2 mg/kg (*p* < 0.0001). Selenium is essential for hepatic antioxidant defense as a cofactor of glutathione peroxidase, and an increase in liver mass may indicate improved metabolic or detoxifying capacity [61]. Nonetheless, supra-physiological selenium levels have been linked to compromised hepatic metabolism and the first indicators of steatosis in other models [62]. Additional investigation—such as evaluating liver enzyme activity, oxidative stress indicators, or histological alterations—is necessary to ascertain if the elevated liver weight signifies advantageous adaptation or early hepatic distress. Consistent with our study, Payne and Southern [63] found that supplementing organic Se resulted in a significant increase in the yields of both breast and leg.

Additionally, MOS supplementation did not affect the carcass-linked traits like the weight of abdominal fat and breast meat production. These outcomes are in line with Pelícia [64], Gomez and Angeles [65], who found that prebiotic inclusion in broilers’ diet didn’t change the yield of breast meat and the percentage of abdominal fat. Likewise, Wang et al. [66] and Shulukh et al. [67] reported that prebiotic provision did not alter the ratio of breast muscle and abdominal fat in broilers. Abdel Hafeez et al. [31] observed similar results and mentioned a non-significant effect of MOS on the carcass traits of the broilers. Prebiotics have been shown to reduce lipid synthesis, but the exact process underlying this effect has not been reported yet. However, it is believed to be due to the presence of beneficial bacteria, such as Lactobacillus, that reduce or inhibit the function of acetyl-CoA carboxylase, which is the rate-limiting enzyme in the synthesis of fatty acids.

### 4.3. Immunity

The immune system is an important element influencing an animal’s performance and general health status. It has been observed that trace elements such as zinc, ferrous, copper, selenium of and manganese play a significant part in maintaining regular immune function and resistance against diseases [68]. Selenium is crucial for the augmentation and strengthening of the immune system, i.e., innate and adaptive immunity [14,69]. Selenium is also well known because it affects biological processes like antioxidant capacity and immunological activities [70]. Se inclusion enhances the humoral immunity and provides an effective immunological response via its effects on the immune cells’ ability to withstand oxidative stress and eliminate free radicals [71]. A lack of Se may cause histopathological alterations in a number of tissues, such as immunity-related organs like the thymus, spleen, and bursa, which would then negatively affect the immune system [72]. The effect of selenomethionine and MOS was found to be significant on the immune organs and ND titers in the reared broilers. Our outcomes inclined with the outcomes of Bakhshalinejad et al. [42], who mentioned a considerable increment in total anti-SRBC titers and IgG levels after dietary intake of organic Se. Additionally, IgG titers improved by 3.49% in response to dietary Se supplementation. Likewise, Cai et al. [72] observed that Se supplementation may boost the IgG and IgM in the serum of broiler birds and suggested 0.3–0.5 mg/kg Se levels in the diet. Likewise, Zamani Moghaddam et al. [73] reported that supplementation with 0.3 mg/kg organic Se in the broiler diet resulted in an optimal production of anti-SRBC titers. When combined, the findings of all the above-mentioned studies indicate that organic forms of selenium supplementation can notably strengthen the cellular and humoral immune systems.

Prebiotics have a vital role in the immunity of broilers, and our findings are in line with the results of Gao et al. [74] who found a linear rise in antibody titer against NDV with the increase in the level of yeast culture in the diet, which indicates that dietary yeast culture supplementation in broiler’ diet has an impact on humoral or systemic immunity. Conversely, Houshmand et al. [75] and Shahir et al. [54] described that prebiotic treatment did not significantly alter the antibody titer against NDV in broilers.

### 4.4. Gut Morphology

Villus height and crypt depth are crucial to ensure the health of the small intestine for appropriate nutrient absorption and preventing translocation of bacteria from the gut [76]. A longer small intestine indicates a healthy gut with more area, which would enhance nutrient absorption and utilization and permit higher cumulative growth. The results of our study agree with the findings of Dalia et al. [77], who found that the broilers fed with organic Se had significantly higher duodenal villus heights, but this was non-significant for the crypt depths. Similarly, Tong et al. [78] described that organic Se in broilers enhanced the intestinal villus height. Nutrient absorption and digestion are the primary roles of the small intestine. It is well recognized that shorter villi result in reduced surface area availability for the absorption of nutrient absorption, and a deeper crypt signifies rapid tissue regeneration [79]. Moreover, villi with shorter height and a deeper crypt depth are associated with increased enterocyte turnover [80]. On the other side, dietary antioxidants may improve the development of enterocytes while shielding them from apoptotic oxidative stress [81]. As a result, the improved villus height observed in our experiment with the higher Se level (although non-significantly) may be due to the ability of organic Se as an external antioxidant agent, which could positively improve the enterocytes’ survival by actively supporting the intestinal glutathione peroxidase (GSH-Px2). All these findings recommend that improvement in retention and assimilation of Se in organic Se-fed birds accounts for improved intestinal integrity and possibly better gut antioxidant status.

Supplementation with MOS resulted in enhanced crypt depth in our study, which agrees with the findings of Yang et al. [49], who also described an increase in crypt depth in broilers fed with MOS. Conversely, Oliveira et al. [82] noted improved morphometrics after the inclusion of MOS in broiler diets and inferred that this may be due to variation in the sources of prebiotics. Moreover, Pelicano et al. [83] stated that the prebiotic-supplemented diet resulted in higher villus height in broilers, as also found in our study. Prebiotic supplementation may reduce the inflammatory process of the intestinal mucosa, which results in improved villus height, secretory functions, digestion and nutrient absorption. Improved villus height is an indication of the beneficial and positive effects of MOS on the broiler chicken gut. Sayrafi et al. [83] also reported comparable results and found an increased VH in response to prebiotics as compared to antibiotics. This may be attributed to the ability of prebiotics to modulate the intestinal microbial communities.

## 5. Conclusions

This experiment confirms the synergistic role of organic selenium and MOS in broiler chickens. The growth performance of broilers was considerably improved with a lower level of Se (0.2 mg/kg) with MOS, whereas the higher organic Se level (0.4 mg/kg) resulted in further enhancement in intestinal morphology, immunological function and carcass quality. All these positive results are believed to be due to the higher bioavailability of organic selenium and enhanced gut health, encouraging better nutrient utilization and immunological resilience. Therefore, the inclusion of 0.4 mg/kg organic Se with 1 g/kg MOS is the best practical approach for broilers. Furthermore, this combination of Se and MOS offers a promising alternative to AGPs, playing its role in the production of sustainable poultry while reducing the risk of antimicrobial resistance.

## Figures and Tables

**Table 1:** Experimental design for the trial.

A 2 × 2 Factorial Arrangement Under CRD		Organic Selenium (Zinc-L-Selenomethionine)	
		Se0.2 mg/kg	Se0.4 mg/kg
**Manan-Oligosaccharide (MOS)**	No MOS added (−)	Straight-run broilers 132 birds, 6 replicates 22 birds/replicate	Straight-run broilers 132 birds, 6 replicates 22 birds/replicate
	MOS at 1.0 g/kg (+)	Straight-run broilers 132 birds, 6 replicates 22 birds/replicate	Straight-run broilers 132 birds, 6 replicates 22 birds/replicate

**Table 2:** Ingredients and nutrient composition of basal diets (% as fed).

Diet Composition	Starter (1–21 d)	Finisher (22–35 d)
**Ingredients**	**%**	**%**
Yellow corn	58.1	61.1
Canola Oil	1.7	2.3
Soybean meal	36.5	33
L-Lysine HCl	0.45	0.4
DL-Methionine	0.23	0.21
L-Threonine	0.15	0.11
Common salt	0.47	0.48
Dicalcium phosphate	0.7	0.5
Limestone	1.6	1.8
Micro mineral premix ^1^*	0.05	0.05
Vitamin premix ^2^	0.05	0.05
**Total**	**100**	**100**
**Analyzed Nutrients (% otherwise noted)**		
Metabolizable Energy (Calculated, Kcal/kg) ^3^	3000	3100
Crude protein	23.00	21.5
Ether extract	5.00	5.00
Crude fiber	5.00	5.00
Crude ash	1.00	1.70
Calcium	0.96	0.87
Available phosphorus	0.48	0.43
Dig-Lysine	1.28	1.15
Dig-Methionine	0.51	1.47
Dig-Threonine	0.86	0.77

^1,2^ Trace minerals and vitamin premix composition were the same as previously described by [36]. * The basal diets contained Se only from the organic (zinc-L-selenomethionine) source. ^3^ ME (KCal/Kg) = ((feed intake × gross energy (GE _diet_)) − (excreta output × GE _excreta_))/feed intake.

**Table 3:** Effects of zinc-L-selenomethionine and MOS on the performance of broiler chickens.

Parameters	Treatments				SEM	*p*-Value		
	Se 0.2 mg/Kg		Se 0.4 mg/kg					
	Se0.2	Se0.2 + MOS	Se0.4	Se0.4 + MOS		Se	MOS	Se × MOS
**FI, g/bird**								
**1–21 d**	1123.03 ^a^	1088.92 ^b^	1111.49 ^a^	1058.74 ^c^	5.44	<0.0001	<0.0001	<0.0001
**22–35 d**	2187.26 ^b^	2027.57 ^d^	2224.10 ^a^	2140.80 ^c^	15.44	<0.0001	<0.0001	<0.0001
**1–35 d**	3310.28 ^b^	3116.49 ^d^	3335.59 ^a^	3199.54 ^c^	18.48	<0.0001	<0.0001	<0.0001
**BWG, g/bird**								
**1–21 d**	846.04 ^c^	900.04 ^a^	867.85 ^b^	895.94 ^a^	5.67	0.232	<0.0001	0.0001
**22–35 d**	1099.75 ^c^	1269.97 ^a^	1190.31 ^b^	1199.65 ^ab^	17.17	0.690	0.002	0.004
**1–35 d**	1945.79 ^c^	2170.00 ^a^	2058.16 ^b^	2095.59 ^b^	20.13	0.430	<0.0001	0.001
**FCR**								
**1–21 d**	1.33 ^a^	1.21 ^c^	1.28 ^b^	1.18 ^c^	0.01	0.006	<0.0001	<0.0001
**22–35 d**	2.00 ^a^	1.60 ^c^	1.87 ^b^	1.79 ^b^	0.04	0.473	<0.0001	0.001
**1–35 d**	1.71 ^a^	1.44 ^d^	1.62 ^b^	1.53 ^c^	0.02	0.901	<0.0001	<0.0001
**Liv, %**								
**1–35 d**	99.09 ^b^	99.85 ^a^	98.94 ^b^	97.39 ^ab^	0.11	0.1181	0.0039	0.0125

FI = feed intake; BWG = body weight gain; FCR = feed conversion ratio; Liv = livability. *p* ≤ 0.05 represents significant difference; SEM = pooled standard error of means. The different superscript letters within a row represent significant differences between the treatmnets

**Table 4:** Influence of zinc-L-selenomethionine and MOS on carcass traits of broilers (35 d).

Parameters	Treatments				SEM	*p*-Value		
	Se 0.2 mg/Kg		Se 0.4 mg/kg					
	Se0.2	Se0.2 + MOS	Se0.4	Se0.4 + MOS		Se	MOS	Se × MOS
**CW, g**	1383.89 ^b^	1285.65 ^c^	1386.91 ^b^	1501.22 ^a^	14.29	<0.0001	0.7417	<0.0001
**CY, %**	65.75 ^bc^	64.77 ^c^	66.72 ^ab^	67.66 ^a^	0.29	0.0006	0.9621	0.0021
**Hrt W, g**	11.31	11.43	11.00	11.43	0.10	0.4466	0.1673	0.3807
**Liv W, g**	46.61 ^b^	45.98 ^b^	49.25 ^a^	49.87 ^a^	0.28	<0.0001	1.0000	<0.0001
**Giz W, g**	42.71 ^b^	43.84 ^b^	45.35 ^a^	46.23 ^a^	0.29	<0.0001	0.0580	<0.0001
**LQW, g**	284.24 ^a^	263.86 ^b^	274.08 ^ab^	290.31 ^a^	2.92	0.1464	0.7095	0.0063
**Br W, g**	529.61 ^b^	485.03 ^c^	544.96 ^b^	602.73 ^a^	7.51	<0.0001	0.5989	<0.0001

CW = carcass weight; CY = carcass yield; Hrt W = heart weight; Liv W = liver weight; Giz W = gizzard weight; LQW = leg quarter weight; BW = breast weight. *p* ≤ 0.05 represents significant difference; SEM = pooled standard error of means. The lowercase superscript letters represent the siginificant differences across means of treatments.

**Table 5:** Impact of zinc-L-selenomethionine and MOS on the immune response of broilers (35 d).

Parameters	Treatments				SEM	*p*-Value		
	Se 0.2 mg/Kg		Se 0.4 mg/kg					
	Se0.2	Se0.2 + MOS	Se0.4	Se0.4 + MOS		Se	MOS	Se × MOS
**Spleen (g)**	2.61 ^d^	2.72 ^c^	2.77 ^b^	2.85 ^a^	0.02	<0.0001	<0.0001	<0.0001
**Bursa (g)**	1.92 ^b^	2.17 ^a^	2.22 ^a^	2.28 ^a^	0.03	0.0002	0.0034	<0.0001

*p* ≤ 0.05 represents significant difference; SEM = pooled standard error of means. The supersscript letters represent significant differences across treatment means.

**Table 6:** Impact of zinc-L-selenomethionine and MOS on duodenal histology of broilers (35 d).

Parameters	Treatments				SEM	*p*-Value		
	Se 0.2 mg/Kg		Se 0.4 mg/kg					
	Se 0.2	Se 0.2 + MOS	Se 0.4	Se 0.4 + MOS		Se	MOS	Se × MOS
**VH**	1525.36	1602.22	1608.00	1737.25	31.19	0.0734	0.0888	0.654
**CD**	278.82	298.33	275.50	304.50	5.91	0.9018	0.0458	0.2268
**VH/CD**	5.57	5.37	5.92	5.71	0.15	0.2764	0.5090	0.6423

VH = villus height; CD = crypt depth; VH/CD = villus height/crypt depth. *p* ≤ 0.05 represents significant difference; SEM = pooled standard error of means.

## Data Availability

The raw data supporting the conclusions of this article will be made available by the authors on request.
